# An Adaptive Elastic Support Seat-Based Magnetorheological Elastomer for Human Body Vibration Reduction

**DOI:** 10.3390/ma17133330

**Published:** 2024-07-05

**Authors:** Wei Ding, Leizhi Wang, Zhaobo Chen, Hongrui Ao, Hui Yan

**Affiliations:** School of Mechatronics Engineering, Harbin Institute of Technology, Harbin 150001, China; 20b908072@stu.hit.edu.cn (W.D.); wangleizhi@hit.edu.cn (L.W.); yanhui@hit.edu.cn (H.Y.)

**Keywords:** adaptive elastic foundation, MRE, vibration reduction, frequency modulation, electromagnetic structure

## Abstract

This paper introduces an electromagnetic structure utilizing the controllable mechanical properties of magnetorheological elastomer (MRE) materials through magnetic flux. An adaptive elastic foundation composed of these materials is explored for vibration reduction and frequency modulation. This study investigates these effects using both a single-mass model and a coupled human–seat model. For objects supported by the adaptive elastic foundation, increasing the magnetic flux enhances the stiffness and damping, thereby significantly reducing the peak response while slightly increasing the resonance frequency. Strategies such as increasing the magnetic flux, reducing the object mass, and minimizing the system’s degrees of freedom and internal damping contribute to enhancing the vibration reduction and frequency modulation in the adaptive elastic foundation. The simulation results indicate that for a seated human (weighing between 72.4 kg and 88.4 kg), the adaptive elastic foundation reduces the head peak response by approximately 15.7% and increases the resonance frequency by approximately 3.4% at a magnetic flux of 138 mT.

## 1. Introduction

In vibrating environments, the human body experiences discomfort [1], which escalates with the increasing human response magnitude [2,3]. Griffin [4] noted that discomfort is minimal in a vibration environment of 0.1 ms^−2^ or less, moderate up to 1 ms^−2^, and significant above 10 ms^−2^. Kjellberg [5] established a positive correlation between discomfort and vibration amplitude over a certain frequency range. Thus, there is a need to mitigate the human response to vibrations for enhanced occupant comfort.

The transfer of vibrations requires the excitation source to be transferred to the human body via the seat. Mitigation strategies can be categorized by the transmission path: one is to reduce the response of the human body, and the other is to mitigate the energy transferred from the excitation source to the seat. The former can reduce the response by altering human–seat coupling [6], but to a limited extent.

There are two methods for mitigating energy transfer. One method is to install vibration-isolation devices to dissipate energy, such as magnetorheological dampers [7]. The other is to alter the resonance frequency by adjusting the floor-to-seat contact stiffness. Reference [8] detailed a magnetorheological damper with variable stiffness, controlled via magnetic fields to switch operation modes and adjust stiffness, thus achieving the effect of frequency modulation (FM). For a seated human, reducing the vibration response through energy dissipation and adjusting the resonance frequency can effectively diminish the response at the original resonance frequency position. The effectiveness of the vibration reduction improves when both methods are applied simultaneously.

Magnetorheological elastomers (MREs) are a family of magnetorheological materials whose properties can be controlled by an external magnetic field, hence the term “smart materials”. Among the properties of MRE materials, the magneto-mechanical properties are the basis for the functional and structural design of their applied devices. Various mathematical and mechanical models have been proposed to explain the magneto-mechanical properties of anisotropic and isotropic MREs. The reviews in [9,10] summarized the different testing methods, fabrication techniques, synthesis methods, and experimental findings of MREs. The review in [9] described several characterization methods for understanding the basic material, fabrication, and functional behavior of MREs. A Review [10] described the factors influencing the magnetorheological (MR) effect of MREs, including the type of base material, type, concentration and distribution of magnetic particles, use of additives, working mode, and magnetic field strength. For specific theoretical models, the magnetically induced mechanics model [11] distinguishes three different forms of spatial distribution of MRE materials, namely, chain, planar, and isotropic; the soft-shell encapsulated nanoparticle model [12] predicts the relationship between the magnetic parameters and the shear modulus; the magneto-mechanical coupling model [13] analyses the effect of prestress on the magnetically induced shear storage modulus; the energy approach [14] describes the constitutive relationship between magnetic induction energy and elastic strain energy; and the magnetoelastic energy storage model [15] takes into account magnetic dipole interactions and finite strains based on the mean deformation gradient and magnetic field microstructure. In addition, there are several studies of linear viscoelastic regions in high-frequency compression [16], studies of magneto-mechanical properties based on compressive [17,18] or tensile [19,20] behaviors, and studies of MREs under uniaxial compression with large strains [21], among others. To describe the magneto-mechanical properties of MREs in the compression mode, the factors influencing the dynamic properties of MREs in the compression mode [22] and their characterization methods [23] were investigated. Special configurations of MREs [24] can have tailored mechanical properties [25] under predetermined compression conditions; therefore, MRE materials are widely used in vibration reduction [26], sensing devices [27], signal monitoring [28], intelligent robotic arms [29], and flexible electronic wearable devices [30]. The properties of MREs in the shear mode have been extensively studied, while MREs with tilt angles in the compression mode have been less studied. Based on magnetic dipole theory and the energy method, one study [31] established an anisotropic MRE magneto-mechanical model by considering the reconfiguration effect of compressive strain on the magnetic chain microstructure, provided a dynamic magneto-mechanical model (including a magnetically induced mechanical model) by robust fitting, and evaluated the stress–strain characteristics of MREs with respect to the tilt angle. Based on a U-shaped electromagnetic structure [32], a columnar electromagnetic structure is established in this paper using MRE materials with a 0° magnetic chain tilt angle. While MREs have been applied in large building structures alongside tuned mass dampers [26], their potential in reducing vibrations in smaller structures like seated humans remains underexplored. The columnar electromagnetic structure presented here, in conjunction with the seat’s original grounding structure, forms an adaptive elastic foundation for vibration reduction and FM in seated humans.

The elastic foundation can adjust the magnetic flux through the current, which affects the properties of the MRE material in order to change the grounding stiffness and damping of the system. This method theoretically allows for the adjustment of the vibration response and resonance frequency in human–seat systems to achieve vibration reduction. Section 2 of this article describes the relationships among the stiffness, damping, and current of electromagnetic structures. The vibration response of a single-degree-of-freedom system with an adaptive elastic foundation is simulated. In Section 3, the current of the electromagnetic structure is adjusted to measure the vibration response of a swept plate supported by an adaptive elastic foundation. This experiment demonstrates the significant vibration reduction ability of the adaptive elastic foundation based on the MRE electromagnetic structure. The variation patterns of the peak response and resonance frequency induced by the adaptive elastic foundation are discussed in Section 4. The adaptive elastic foundation is introduced into the seated human body model, and the transfer ratios of the floor to each part of the human body are simulated. Due to the human body’s higher mass and damping, the peak reduction ratio and FM ratio of the transfer ratios are smaller compared to those of a single-mass plate.

## 2. Simulation Calculation

### 2.1. Stiffness and Damping of the MRE Electromagnetic Structure

**Figure 1:**
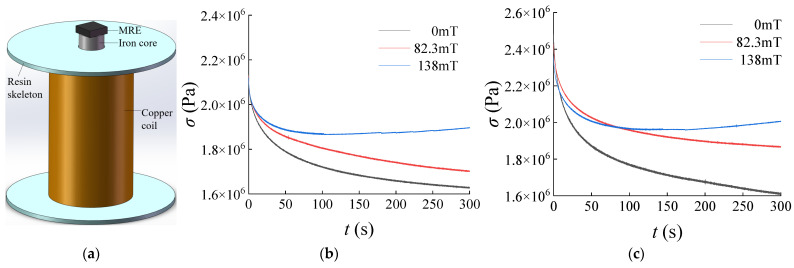
Effect of magnetic flux on stress relaxation characteristics of anisotropic MRE (*α* = 0°, *ε* = 10%): (**a**) MRE electromagnetic structure; (**b**) 60% by mass of CIP; (**c**) 80% by mass of CIP.

An electromagnetic structure capable of adjusting the stiffness and damping of an MRE material was constructed. The MRE material used in the electromagnetic structure had dimensions of 16 mm × 16 mm × 5 mm, as depicted in Figure 1a. By energizing the copper coil, a magnetic field is generated along the direction of the core, thereby changing the stiffness and damping characteristics of the MRE material.

Two types of anisotropic MRE materials were used for testing according to the mass percentage of carbonyl iron particles (CIP), namely, 60% and 80%. The magnetic chain inclination angle *α* of the MRE was 0. The strain *ε* at the height of the MRE material was kept at 10% by applying pressure on the MRE material at different magnetic fluxes. The duration of each test was 300 s. The variation in stress with time in the MRE material is shown in Figure 1.

**Figure 2:**
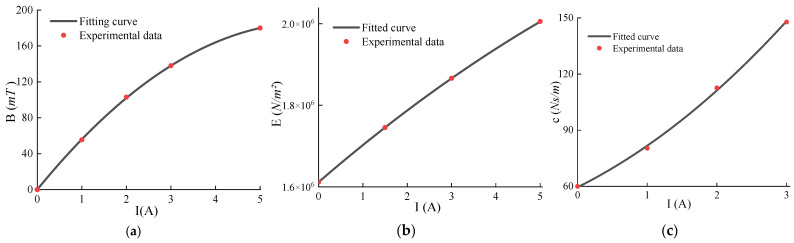
Magneto-mechanical properties of MRE materials at 10% strain with different currents: (**a**) magnetic flux at the corresponding current; (**b**) magnetically induced modulus at the corresponding current; (**c**) damping at the corresponding current.

The test results indicated that the stress levels in the MRE material decreased rapidly and then stabilized with increasing the vibration duration. The stress variation range for the MRE with an 80% CIP mass percentage was notably larger compared to that with a 60% CIP mass percentage. Subsequent measurements therefore focused on the MRE with an 80% CIP mass fraction. The experimental curves demonstrate that the magneto-mechanical properties of the MRE reached a near-stable state around 300 s. The fitted curves depicting the magnetic flux, magneto-induced modulus, and damping of the MRE material measured at different currents under 10% strain are shown in Figure 2.

It was assumed that the magnetic flux *B*, magneto-induced modulus *E*, and damping *c* all conformed to a second-order polynomial relationship with the current *I*:

(1) $$T=a_{1}I^{2}+a_{2}I+a_{3},T=\left\{B,E,c\right\}$$

The least squares method was used to fit the experimental data separately with the following fitting equations:

(2) $$min\;F=\sum_{1}^{n}{\left|\left|T_{\left(i\right)}^{\prime }\right|-\left|T_{\left(i\right)}\right|\right|}^{2}$$

$T_{i}^{\prime }$—The experimental value at the corresponding current;

$T_{i}$—The theoretical value at the corresponding current.

The results of coefficient fitting are displayed in Table 1:

The stiffness of the MRE electromagnetic structure was calculated as follows:

(3) $$k_{MRE}=n_{1}EA/h$$

where $n_{1}$ is the number of MRE electromagnetic structures, *E* is the magneto-induced modulus, *A* is the support area, and *h* is the thickness.

### 2.2. Vibrating Seat Model

**Figure 3:**
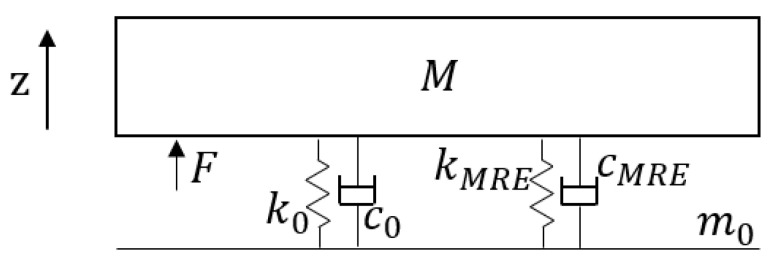
Single-mass seat vibration model.

A seat within a vibration environment was modeled as a single-degree-of-freedom system, illustrated in Figure 3. The seat system was simplified as a single-mass model with an assumed mass of 10 kg. The contact stiffness between the seat and the floor was modeled using the parallel stiffness $k_{0}$ of four standard springs, specified as 3.5 × 35 × 90 GB20892009 [33], with the effective number of spring coils set to 5.5. The contact damping $c_{0}$ between the seat and the floor was assumed to be 4 × 20 Nm/s [34]. The MRE electromagnetic structure provided support to the seat with stiffness $k_{MRE}$ and damping $c_{MRE}$. Together with the original grounding structure, it formed an adaptive elastic foundation for the seat.

The linear design equation for the standard spring stiffness is as follows:

(4) $$k_{s}=\frac{Gd^{4}}{8D^{3}n_{2}}$$

where *G* is the shear modulus of the spring (78,800 MPa), *d* is the wire diameter (m), *D* is the mid diameter (m), and $n_{2}$ is the effective number of coils.

The seat-to-floor contact stiffness and damping without the MRE electromagnetic structure are as follows:

(5) $$K=k_{0}=4k_{s},\;C=c_{0}$$

The seat-to-floor contact stiffness and damping when using a 4-group MRE electromagnetic structure are as follows:

(6) $$K=k_{0}+k_{MRE},\;C=c_{0}+c_{MRE}$$

### 2.3. Calculation Results

The equation of motion for the seating system is as follows:

(7) $$M\ddot{z}+C\dot{z}+Kz=F$$

where *M* is the total mass of the seating system, *C* is the total damping, *K* is the total stiffness, and *F* is the external excitation.

**Figure 4:**
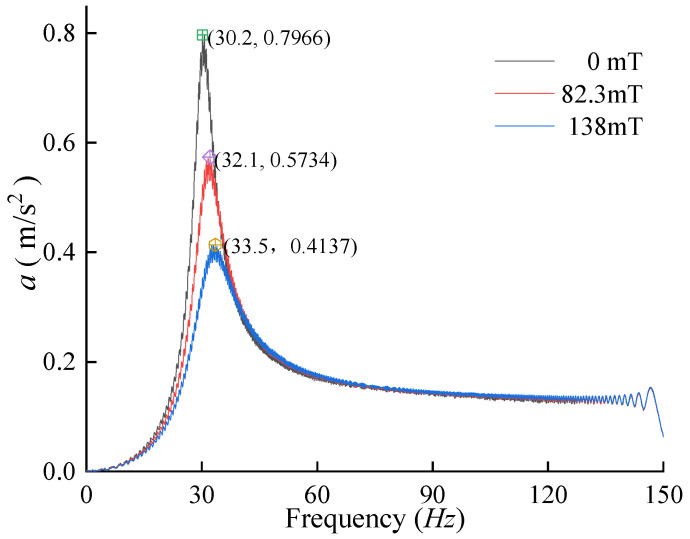
Spectrogram of the swept response of the single-degree-of-freedom system for different fluxes.

Currents of 0 A, 1.5 A, and 3 A were applied to the MRE electromagnetic structure, corresponding to magnetic fluxes of 0 mT, 80.3 mT, and 138 mT, respectively. A swept excitation *F* was applied to the model with a frequency range of 0.1 Hz to 150 Hz over a duration of 300 s. The spectrogram of the swept frequency response is shown in Figure 4.

At 0 mT magnetic flux, the peak response of the system was 0.7966 m/s^2^ with a resonance frequency of 30.2 Hz. With 82.3 mT magnetic flux, the peak response decreased to 0.5734 m/s^2^, and the resonance frequency increased to 32.1 Hz, marking a 28.02% reduction in peak response and a 1.9 Hz rise in resonance frequency compared to the no-current condition. At 138 mT magnetic flux, the peak response further decreased to 0.4137 m/s^2^, and the resonance frequency increased to 33.5 Hz. Compared to the 0 A current condition, this represents a 48.07% decrease in peak response and a 3.3 Hz increase in resonance frequency.

## 3. Vibration Experiment

The MRE electromagnetic structure was applied into an adaptive elastic foundation to assess the vibration reduction and frequency modulation effects through measurement of the vibration response of an excited object.

### 3.1. Experimental Equipment

**Figure 5:**
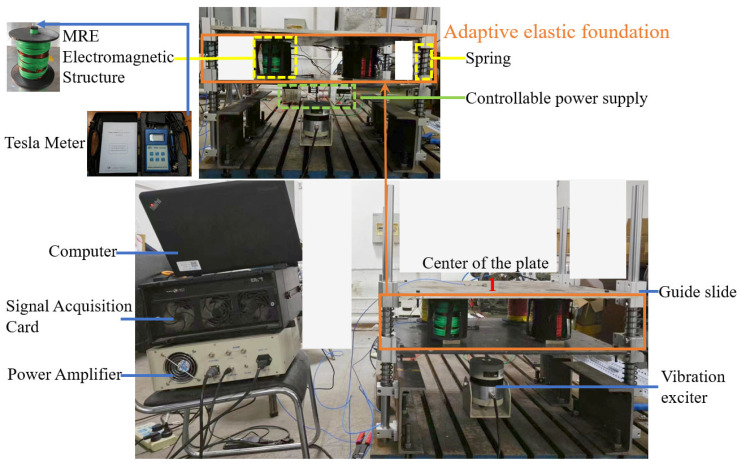
Experimental equipment for analyzing the vibration reduction ability of the adaptive elastic foundation.

As shown in Figure 5, the experimental setup included mainly two metal plates, where the upper plate functioned as the study subject. Meanwhile, the lower plate simulated floor vibrations through a vibration exciter. To ensure directional control of the upper plate’s displacement, it was connected to four vertical struts via guide sliders. Positioned between these plates were four sets of MRE electromagnetic structures coupled with springs, collectively forming an adaptive elastic foundation. To maintain an approximate 10% vertical strain of the MRE material during resting conditions, we carefully selected the springs for both the upper plate (2.5 × 35 × 90 GB20892009) and the overall support structure (3.5 × 35 × 90 GB20892009). The vibrating mass of the upper plate weighed 6.4 kg, and its response was measured using a B&K accelerometer (4507B; Spectris Instrumentation and Systems Shanghai Ltd. Shanghai Branch, Unit 401, Building 9, No. 99 Tianzhou Road, Shanghai, China; 9.949 mV/ms^−2^). This accelerometer was placed at position 1, as indicated in Figure 5. The magnetic flux within the MRE structures was controlled by a controllable power supply and monitored using a Tesla Meter (HT201/HT208; ShangHai HengTong Magnetoelectricity Co., Ltd., Shanghai, China; 0.01 mT/0.1 mT). The experimental rig employed a swept-frequency excitation force applied via a vibration exciter, while data acquisition was efficiently managed through a combination of a computer, a signal acquisition card, and a power amplifier.

### 3.2. Results

A sweeping excitation of 360 s was applied to the lower plate, and the response of the upper plate was measured under each of the following three operating conditions:

1. No current in the MRE electromagnetic structure;
2. MRE electromagnetic structure at a current of 3 A with a magnetic flux of 138 mT;
3. MRE electromagnetic structure at a current of 5 A with a magnetic flux of 180 mT.

**Figure 6:**
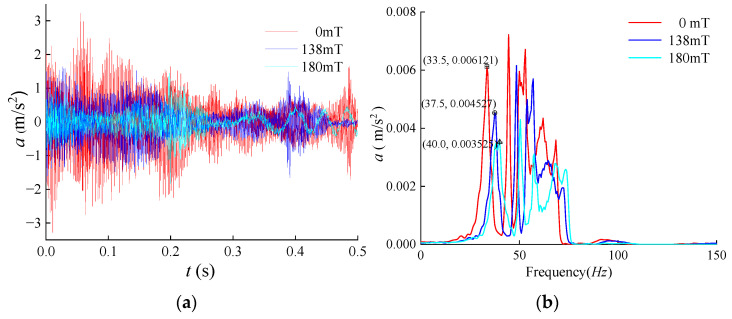
Experimental vibration response at different magnetic fluxes: (**a**) time domain diagram; (**b**) spectrogram.

The responses for the three sets of conditions are shown in Figure 6.

In the experimentally measured response, several groups of peaks were observed, with the first four groups following a consistent pattern: as the magnetic field strength gradually increased, there was a noticeable attenuation in the vibration response, accompanied by a slight increase in the resonance frequency.

The resonance frequency of the experimentally measured first-order response closely matched the simulation results. Specifically, at a magnetic flux of 0 mT, the peak first-order resonance response was 0.006121 m/s^2^ with a resonance frequency of 33.5 Hz. At 138 mT, the peak first-order response decreased to 0.004527 m/s^2^, and the resonance frequency increased to 37.5 Hz, indicating a 26.04% decrease in the peak value and a 4 Hz increase in the resonance frequency compared to the no-current condition. At 138 mT, the peak first-order response further decreased to 0.003525 m/s^2^, and the resonance frequency increased to 40 Hz compared to the 0 A current condition. This represents a 42.41% reduction in the peak value and a 6.5 Hz increase in the resonance frequency.

## 4. Discussion

### 4.1. Analysis of Vibration Reduction

Both the simulations and experimental results indicate that increasing the magnetic flux of the adaptive elastic foundation reduced the system’s response amplitude significantly while slightly increasing the resonant frequency. At a magnetic flux of 138 mT, there existed a certain disparity between the simulation calculations and the experimental findings. The possible explanations for this include the following:

(1) Despite efforts in the experiment to allow the internal stress of the MRE to stabilize before measuring vibration response, Figure 1 shows that the stress curves of the MRE under a magnetic field stabilized only to a certain extent. The rubber softened under pressure, and the material hardened due to CIP under the magnetic field, but these effects could not reach equilibrium in the vibrating environment, resulting in dynamic changes in the actual stiffness and damping of the adaptive elastic foundation.
(2) The experimental setup’s structure deviated from a purely single-mass configuration; the guide sliders introduced additional rolling friction, dissipating some energy. Moreover, slight angular deviations in the I-beam folding angle from 90° caused vertical misalignment of the four strut rails, resulting in discrepancies between the actual and simulated stiffness and damping.
(3) The post-processing software used for analyzing the experimental spectrograms employed a frequency resolution of 0.5 Hz, which may have led to an inadequate frequency resolution.

In conclusion, modulation of the magnetic flux through an electrical current in an adaptive elastic foundation can alter the stiffness and damping of the MRE electromagnetic structure, thereby influencing the response and resonance frequency of the vibrating object.

### 4.2. Vibration Reduction in a Seated Human Body

**Figure 7:**
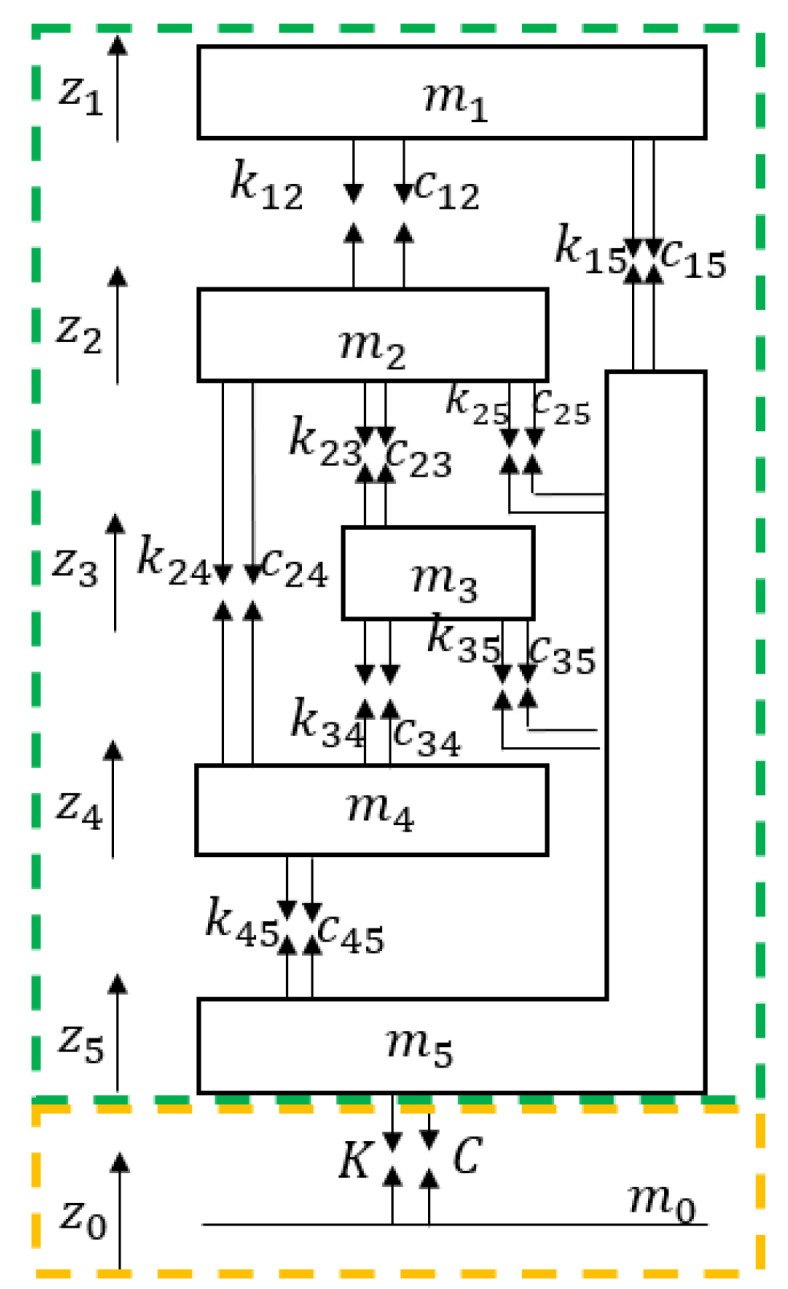
Coupled human–seat model with adaptive elastic foundation support (green frame indicates human-seat coupling model; yellow frame indicates adaptive elastic foundation).

Reference [6] investigated the vibration response under different coupling modes between humans and seats, finding that the optimal response reduction occurred with full contact between the human and the backrest, as depicted in the green frame of Figure 7. An adaptive elastic foundation was loaded to the bottom of the seat to study the vibration reduction provided to a seated human, as shown in the yellow frame of Figure 7. In the model, $m_{1}$~$m_{5}$ represent the masses of the head, chest, abdomen, pelvis, and seat, respectively. The seat supported an upper body mass of 60.67 kg, resulting in a total human mass of 82.4 kg [35]. These masses were interconnected by stiffness and damping elements. $m_{0}$ represents the floor, which is massless. $z_{0}$~$z_{5}$ denote the vertical displacements corresponding to each mass. The mass of the seat $m_{5}$ was weighed as 32.4 kg. Some parameters from Wei’s model [6] are listed in Table 2. The ground stiffness K and damping C of the seat adhered to previously established values.

When the seated human–seat coupled system was excited by the displacement of the floor, the biodynamic model equations of the motion of the system were as follows:

(8) $$\left\{ \begin{array}{l} m_{1}{\ddot{z}}_{1}+k_{12}(z_{1}-z_{2})+c_{12}({\dot{z}}_{1}-{\dot{z}}_{2})+k_{15}(z_{1}-z_{5})+c_{15}({\dot{z}}_{1}-{\dot{z}}_{5})=0\\ m_{2}{\ddot{z}}_{2}+k_{12}(z_{2}-z_{1})+k_{23}(z_{2}-z_{3})+k_{24}(z_{2}-z_{4})+k_{25}(z_{2}-z_{5})+c_{12}({\dot{z}}_{2}-{\dot{z}}_{1})+c_{23}({\dot{z}}_{2}-{\dot{z}}_{3})+c_{24}({\dot{z}}_{2}-{\dot{z}}_{4})+c_{25}({\dot{z}}_{2}-{\dot{z}}_{5})=0\\ m_{3}{\ddot{z}}_{3}+k_{23}(z_{3}-z_{2})+k_{34}(z_{3}-z_{4})+k_{35}(z_{3}-z_{5})+c_{23}({\dot{z}}_{3}-{\dot{z}}_{2})+c_{34}({\dot{z}}_{3}-{\dot{z}}_{4})+c_{35}({\dot{z}}_{3}-{\dot{z}}_{5})=0\\ m_{4}{\ddot{z}}_{4}+k_{24}(z_{4}-z_{2})+k_{34}(z_{4}-z_{3})+k_{45}(z_{4}-z_{5})+c_{24}({\dot{z}}_{4}-{\dot{z}}_{2})+c_{34}({\dot{z}}_{4}-{\dot{z}}_{3})+c_{45}({\dot{z}}_{4}-{\dot{z}}_{5})=0\\ m_{5}{\ddot{z}}_{5}+k_{15}(z_{5}-z_{1})+k_{25}(z_{5}-z_{2})+k_{35}(z_{5}-z_{3})+k_{45}(z_{5}-z_{4})+K_{50}(z_{5}-z_{0})+c_{15}({\dot{z}}_{5}-{\dot{z}}_{1})+c_{25}({\dot{z}}_{5}-{\dot{z}}_{2})+c_{35}({\dot{z}}_{5}-{\dot{z}}_{3})+c_{45}({\dot{z}}_{5}-{\dot{z}}_{4})+C_{50}({\dot{z}}_{5}-{\dot{z}}_{0})=0 \end{array}\right.$$

Equation (8) is summarized in matrix form as follows:

(9) $${\left[M\right]}_{5\times 5}{\left\{\ddot{z}\right\}}_{5\times 1}+{\left[C\right]}_{5\times 5}{\left\{\dot{z}\right\}}_{5\times 1}+{\left[K\right]}_{5\times 5}{\left\{z\right\}}_{5\times 1}={\left\{f_{z}\right\}}_{5\times 1}$$

where $\left[M\right]$, $\left[C\right]$, and $\left[K\right]$ are the 5 × 5 mass, damping, and stiffness matrices, respectively; $\ddot{z}$, $\dot{z}$, and z are the acceleration, velocity, and displacement vectors of the mass block, respectively; and $f_{z}$ is the force vector generated by the floor displacement.

$$M=\left[\begin{array}{ccccc} m_{1} & 0 & 0 & 0 & 0\\ 0 & m_{2} & 0 & 0 & 0\\ 0 & 0 & m_{3} & 0 & 0\\ 0 & 0 & 0 & m_{4} & 0\\ 0 & 0 & 0 & 0 & m_{5} \end{array}\right],$$

$$K=\left[\begin{array}{ccccc} k_{12}+k_{15} & -k_{12} & 0 & 0 & -k_{15}\\ -k_{12} & k_{12}+k_{23}+k_{24}+k_{25} & -k_{23} & -k_{24} & -k_{25}\\ 0 & -k_{23} & k_{23}+k_{34}+k_{35} & -k_{34} & -k_{35}\\ 0 & -k_{24} & -k_{34} & k_{24}+k_{34}+k_{45} & -k_{45}\\ -k_{15} & -k_{25} & -k_{35} & -k_{45} & k_{15}+k_{25}+k_{35}+k_{45}+K \end{array}\right],$$

$$C=\left[\begin{array}{ccccc} c_{12}+c_{15} & -c_{12} & 0 & 0 & -c_{15}\\ -c_{12} & c_{12}+c_{23}+c_{24}+c_{25} & -c_{23} & -c_{24} & -c_{25}\\ 0 & -c_{23} & c_{23}+c_{34}+c_{35} & -c_{34} & -c_{35}\\ 0 & -c_{24} & -c_{34} & c_{24}+c_{34}+c_{45} & -c_{45}\\ -c_{15} & -c_{25} & -c_{35} & -c_{45} & c_{15}+c_{25}+c_{35}+c_{45}+C \end{array}\right],$$

$$f_{z}=\left[\begin{array}{c} 0\\ 0\\ 0\\ 0\\ Kz_{0}+C{\dot{z}}_{0} \end{array}\right].$$

Equation (9) is solved using the frequency domain method, starting with the Laplace transform:

(10) $$S^{2}\left[M\right]\left\{Z\left(s\right)\right\}+S\left[C\right]\left\{Z\left(s\right)\right\}+\left[K\right]\left\{Z\left(s\right)\right\}=\left[B\right]\left[\begin{array}{c} 1\\ s \end{array}\right]Z_{0}\left(s\right)$$

where $B=\left[\begin{array}{cc} 0 & 0\\ 0 & 0\\ 0 & 0\\ 0 & 0\\ K & C \end{array}\right]$.

If $\left[A\right]=S^{2}\left[M\right]+S\left[C\right]+\left[K\right]$, then the transfer rate from the floor to each mass block is as follows:

(11) $$H\left(s\right)=\frac{Z\left(s\right)}{Z_{0}\left(s\right)}={\left[A\right]}^{-1}\left[B\right]\left[\begin{array}{c} 1\\ s \end{array}\right]$$

Let *s* = *jω*; then, the floor-to-head transfer ratio (*FTHT*), floor-to-chest transfer ratio (*FTCT*), floor-to-abdomen transfer ratio (*FTAT*), and floor-to-pelvis transfer ratio (*FTPT*) are as follows:

(12) $$\left\{\begin{array}{l} FTHT=\frac{z_{1}(j\omega )}{z_{0}(j\omega )}\\ FTCT=\frac{z_{2}(j\omega )}{z_{0}(j\omega )}\\ FTAT=\frac{z_{3}(j\omega )}{z_{0}(j\omega )}\\ FTPT=\frac{z_{4}(j\omega )}{z_{0}(j\omega )} \end{array}\right.$$

**Figure 8:**
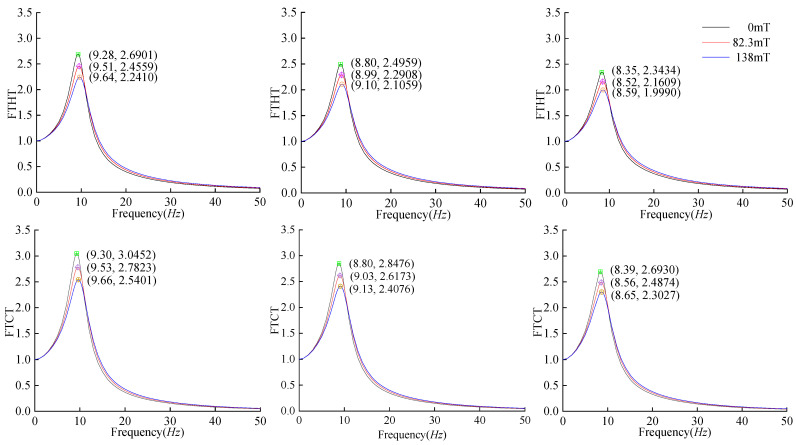

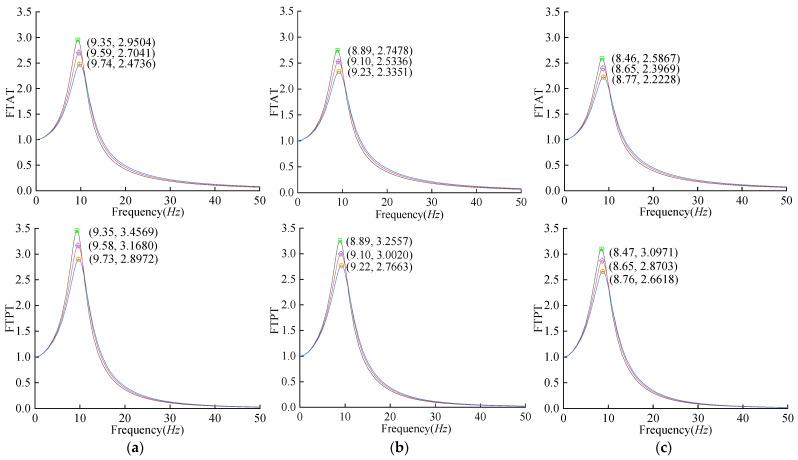
Transfer ratios at different magnetic fluxes for three human body weights: (**a**) body mass coefficient of 0.9; (**b**) body mass coefficient of 1.0; and (**c**) body mass coefficient of 1.1.

For Wei’s model, the simulation results from Figure 8b indicate that at 0 mT magnetic flux, the peak transfer ratios from the floor to the head, chest, abdomen, and pelvis were 2.4959, 2.8476, 2.7478, and 3.2557, respectively, with resonance frequencies of 8.80 Hz, 8.80 Hz, 8.89 Hz, and 8.89 Hz, respectively. At 82.3 mT magnetic flux, the peak transfer ratios from the floor to the head, chest, abdomen, and pelvis were 2.2908, 2.6173, 2.5336, and 3.0020, with resonance frequencies of 8.99 Hz, 9.03 Hz, 9.10 Hz, and 9.10 Hz, respectively. Upon reaching 138 mT magnetic flux, the peak transfer ratios from the floor to the head, chest, abdomen, and pelvis were 2.1059, 2.4076, 2.3351, and 2.7663, with resonance frequencies of 9.10 Hz, 9.13 Hz, 9.23 Hz, and 9.22 Hz, respectively. Compared to the single-mass model, the coupled model exhibited reduced peak response ratios alongside lower resonance frequencies and FM ratios. To discuss the influence of mass on the vibration and FM, the model mass of Wei [6] was considered as the initial human mass. The mass was multiplied by coefficients of 0.9 and 1.1 (resulting in a total human mass of 72.4 kg and 88.4 kg, respectively), with the corresponding transfer ratio curves shown in Figure 8a and Figure 8c, respectively.

Table 3 summarizes the peak and resonance frequency variations in the transfer ratios from Wei’s model supported by an adaptive elastic foundation at different magnetic fluxes. At a constant mass, a higher magnetic flux led to lower peak transfer ratios and higher resonance frequencies, indicating better vibration reduction for the adaptive elastic foundations. The peak vibration reduction ratio decreased slightly from top to bottom across all body parts, with the head experiencing the smallest reduction amplitude but the highest proportional decrease. At 138 mT magnetic flux, the adaptive elastic foundation reduced the peak response of the seated human body by approximately 15.3% and increased the resonance frequency by approximately 3.7%.

Table 4 displays the variations in the peak *FTHT* ratios and resonance frequencies for the three different body weights under various magnetic fluxes. A lower body mass corresponded to greater changes in the peak transfer ratio and resonance frequency, suggesting enhanced vibration reduction by the adaptive elastic foundation. For seated humans weighing between 72.4 kg and 88.4 kg, the adaptive elastic foundation at 138 mT magnetic flux reduced the peak head response by approximately 15.7% and increased the resonance frequency by approximately 3.4%.

The comprehensive analysis of Table 3 and Table 4 reveals that under constant magnetic flux, the smaller the human body mass, the greater the peak and resonance frequencies of the transfer ratio and the more significant the variations in the peak and resonance frequencies due to changes in the magnetic flux. This phenomenon can be explained as follows: when the initial human mass is multiplied by a mass coefficient ‘*a*’, the equation becomes $\left[A\right]=S^{2}\left[M\right]/a+S\left[C\right]+\left[K\right]$. As ‘*a*’ decreases, H(s), representing the transfer ratio in Equation (11), increases, leading to a more pronounced change in the transfer ratio due to mass variation. The smaller the mass of the vibrating object, the higher the resonance frequency √(k/m), and the more significant the frequency changes due to stiffness variations.

When the mass coefficient was set to 0.166, corresponding to a 10 kg human, the calculations show that the peak *FTHT* at a 138 mT magnetic flux was 40.75% lower than at 0 mT, slightly less than the 48.07% peak reduction for the 10 kg single-mass model. The vibration reduction for the human–seat coupling model with the same mass was weaker than for the single-mass model. This is because the human body, with its multiple degrees of freedom and significant damping, is more flexible and structurally complex than a single-mass block. Compared with the damping of the human–seat coupling system, the incremental damping of the adaptive elastic foundation from an increased magnetic flux was minimal. Consequently, for vibration reduction purposes, the human–seat coupling system, with its greater flexibility and complex structure, was less effective than the lighter single-mass model.

In summary, an adaptive elastic foundation effectively reduces vibrations for seated humans. It diminishes the peak responses and slightly raises the resonance frequencies. Enhancing the magnetic field strength increases the stiffness and damping of MRE electromagnetic structures, while increasing the prestrain ε of the MRE material improves the damping under zero-magnetic-field conditions. Both approaches can broaden the tuning range of the magneto-mechanical properties of the adaptive elastic foundation, thereby enhancing the vibration reduction capabilities of the human–seat system.

## 5. Conclusions

The effectiveness of vibration reduction through adjustments in the coupling mode between the human body and seat is constrained. In a human–seat coupling system, modifying the energy consumption and adjusting the resonance frequency can diminish the responses at the original resonance frequencies. This study proposes a vibration reduction approach by integrating an adaptive elastic foundation into the seat, utilizing magnetic flux to control the stiffness and damping of the foundation. This adjustment alters the response and resonance frequency of the vibration object. Specifically, increasing the magnetic flux enhances both the stiffness and damping of the adaptive elastic foundation, thereby reducing the vibration response and increasing the resonance frequency.

Rubber softens under pressure, while CIP hardens under magnetic fields. Achieving a state of perfect equilibrium between them is almost impossible. Therefore, the stiffness and damping of the adaptive elastic foundation will change dynamically. For vibrating objects, under identical magnetic flux, lighter masses exhibit higher peak transfer ratios and resonance frequencies. Furthermore, increasing the magnetic flux yields greater reductions in the peak transfer ratio, and the FM amplitude increases with a decreased object mass, enhancing the vibration reduction. Similarly, at constant mass, a higher magnetic flux decreases the peak transfer ratio and increases the FM ratio, enhancing the vibration reduction, particularly for structurally simpler and less flexible objects.

This research demonstrates that an adaptive elastic-foundation-supported human–seat coupling system reduces the peak *FTHT* by approximately 15.7% and increases the resonant frequency by approximately 3.4% under a 138 mT magnetic flux. An adaptive elastic foundation effectively manages the vibration response of seated humans in vibrating environments, achieving the FM of resonant frequencies. This study offers insights for designing adaptive elastic foundations to mitigate seat vibrations across various applications.

## Figures and Tables

**Table 1:** Polynomial coefficients.

	$a_{1}$	$a_{2}$	$a_{3}$
*B*	−5	61	0
*E*	−3026	93,820	1,612,000
*c*	3.7	18.46	59.56

**Table 2:** Human–seat biodynamic model parameters [6].

Mass/(kg)					Stiffness/(10^3^ N/m)								Damping/(10^2^ Ns/m)							
$m_{1}$	$m_{2}$	$m_{3}$	$m_{4}$	$m_{5}$	$k_{12}$	$k_{23}$	$k_{34}$	$k_{45}$	$k_{24}$	$k_{15}$	$k_{25}$	$k_{35}$	$c_{12}$	$c_{23}$	$c_{34}$	$c_{45}$	$c_{24}$	$c_{15}$	$c_{25}$	$c_{35}$
4.170	15.00	5.500	36.00	32.40	134.4	10.00	20.00	49.34	192.0	49.34	49.34	49.34	2.500	2.00	3.300	24.75	9.091	24.75	24.75	24.75

**Table 3:** Changes in the peak transfer ratio and resonance frequency of Wei’s model at different magnetic fluxes.

Body Part		Absolute				Percentage			
		Head	Chest	Abdomen	Pelvis	Head	Chest	Abdomen	Pelvis
Peak reduction	82.3 mT	0.2051	0.2303	0.2142	0.2537	8.217%	8.088%	7.795%	7.792%
	138 mT	0.3900	0.4400	0.4127	0.4894	15.63%	15.45%	15.02%	15.03%
Increase in frequency	82.3 mT	0.19	0.23	0.21	0.21	2.2%	2.6%	2.4%	2.4%
	138 mT	0.30	0.33	0.34	0.33	3.4%	3.8%	3.8%	3.7%

**Table 4:** *FTHT* peak vibration reduction for three human masses at different fluxes.

Mass Coefficient		Absolute			Percentage		
		0.9	1.0	1.1	0.9	1.0	1.1
Peak reduction	82.3 mT	0.2342	0.2051	0.1825	8.706%	8.217%	7.788%
	138 mT	0.4491	0.3900	0.3444	16.69%	15.63%	14.70%
Increase in frequency	82.3 mT	0.23	0.19	0.17	2.5%	2.2%	2.0%
	138 mT	0.36	0.30	0.24	3.9%	3.4%	2.9%

## Data Availability

The original contributions presented in the study are included in the article, further inquiries can be directed to the corresponding authors.
